# The effect of general practice team composition and climate on staff and patient experiences: a systematic review

**DOI:** 10.3399/BJGPO.2023.0111

**Published:** 2024-02-21

**Authors:** Ruth Abrams, Bridget Jones, John Campbell, Simon de Lusignan, Stephen Peckham, Heather Gage

**Affiliations:** 1 School of Health Sciences, University of Surrey, Guildford, UK; 2 Surrey Health Economics Centre, Department of Clinical and Experimental Medicine, University of Surrey, Guildford, UK; 3 University of Exeter Medical School, University of Exeter, Exeter, UK; 4 Nuffield Department of Primary Care Health Sciences, University of Oxford, Oxford, UK; 5 Centre for Health Services Studies, University of Kent, Canterbury, UK

**Keywords:** general practice, teams, composition, climate, staff, patients

## Abstract

**Background:**

Recent policy initiatives seeking to address the workforce crisis in general practice have promoted greater multidisciplinarity. Evidence is lacking on how changes in staffing and the relational climate in practice teams affect the experiences of staff and patients.

**Aim:**

To synthesise evidence on how the composition of the practice workforce and team climate affect staff job satisfaction and burnout, and the processes and quality of care for patients.

**Design & setting:**

A systematic literature review of international evidence.

**Method:**

Four different searches were carried out using MEDLINE, Embase, Cochrane Library, CINAHL, PsycINFO, and Web of Science. Evidence from English language articles from 2012–2022 was identified, with no restriction on study design. Preferred Reporting Items for Systematic Reviews and Meta-Analyses (PRISMA) guidelines were followed and data were synthesised thematically.

**Results:**

In total, 11 studies in primary healthcare settings were included, 10 from US integrated healthcare systems, one from Canada. Findings indicated that when teams are understaffed and work environments are stressful, patient care and staff wellbeing suffer. However, a good relational climate can buffer against burnout and protect patient care quality in situations of high workload. Good team dynamics and stable team membership are important for patient care coordination and job satisfaction. Female physicians are at greater risk of burnout.

**Conclusion:**

Evidence regarding team composition and team climate in relation to staff and patient outcomes in general practice remains limited. Challenges exist when drawing conclusions across different team compositions and definitions of team climate. Further research is needed to explore the conditions that generate a ‘good’ climate.

## How this fits in

The review findings are relevant to the current workforce pressures in general practice. They demonstrate that how well a practice team works together affects staff wellbeing and patient care. A good relational team climate can mitigate against the adverse effects on staff and patients of high workloads. Implications for general practice are explored.

## Introduction

Even before the global COVID-19 pandemic in 2020, general practice in the UK was facing a workforce crisis.^1^ The number of full-time equivalent (FTE) GPs was falling while workloads were increasing owing to population ageing and increased prevalence of long-term conditions.^2,3^ Policy initiatives have included the introduction of new roles into general practice,^4,5^ but evidence is lacking on what team composition works best for staff and patients. The environment in which employees interact on a daily basis (team climate), however, also affects care delivery,^6,7^ staff wellbeing, and job satisfaction.^8,9^ As independent contractors, general practices make their own staffing decisions and manage their own teams. To inform organisational decision making, we conducted a systematic review to identify evidence on how team composition and team climate in general practice affect outcomes for staff and patients.

## Method

### Conceptual framework

**Figure 1.**
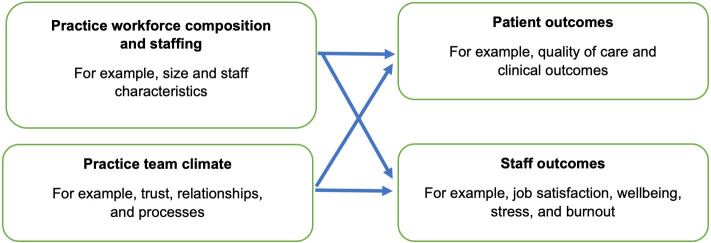
Conceptual framework

Guided by a conceptual framework (Figure 1), this review asked: how 1) the composition, and 2) the climate of a general practice team impacts on the outcomes for a) its staff, and b) its patients.

For the purposes of this review, we define a general practice team as involving ≥2 types of staff, including non-clinical managerial and administrative staff.^10^ Team composition reflects structure, including all professions, grades, age, and sex of staff.^11^ Team climate refers to the relational processes of teamworking, including shared perceptions of organisational policies, practices, and procedures, along with psychosocial aspects such as trust.^11^ Although influenced by the underlying organisational culture,^12^ team climate is generally considered something that is more easily manipulated by team leaders to promote productivity.^13^ Team climate has been equated with team functioning.^14^ Culture is a deeper and more engrained concept, reflecting an organisations’ norms of behaviour, beliefs, and values.^15^ A range of outcomes for staff and patients arising from differences or changes in team composition and team climate were of interest, including staff job satisfaction, wellbeing, stress or burnout, and quality of care for patients.

### Search strategy

A four-stage iterative search was carried out between December 2021 and March 2022. Search results were uploaded into Rayyan software^16^ for screening and quality assessment (Table 1). The Preferred Reporting Items for Systematic Reviews and Meta-Analyses (PRISMA) checklist for reporting transparency for systematic reviews were followed.^17^

**Table 1.** Summary of review methodology

**Review methodology**		
Search	Databases	· MEDLINE, Embase, Cochrane Library, CINAHL, PsycINFO, and Web of Science
The four search strategies can be found in Supplementary Boxes S1–S4. The associated PRISMA diagrams are in Supplementary Figures S1–S4	Terms	· Terms related to primary health care (such as family practice and general practice) and teams (including, but not limited to, staff, interprofessional, interdisciplinary, and multidisciplinary).
	Limiters search stage 1	· Date: January 2015 to December 2021 · Language: English only, because of resource limitations · Countries: where systems of health care were comparable to the UK, such as Canada, New Zealand, and Australia, and excluding studies set in low- and middle-income countries (because of different levels of resources and priorities), and in the US (because of its heterogenous system of provision, dominance of private insurance funding, and lack of universal coverage).
	Search stage 2	· US only. Studies set in US integrated care systems (that align enrolled patients with primary healthcare practitioners and use gatekeeping to specialist services) added because search 1 returned four US articles (despite the country filters) that were considered relevant. Search 2 was same as search 1 in all other respects.
	Search stage 3	· Targeted search, including additional keywords that searches 1 and 2 had identified as potentially relevant, including: ’characteristics’ or ‘structure’ or ‘ratio’ or ‘size’.
	Search stage 4	· Date range extended back to January 2012 because searches 1–3 had identified relevant earlier articles outside the original search dates. Stage 4 was in 2 steps: search criteria 1 and 2 combined, and search criteria 3.
Screening	Titles and abstracts, followed by full text	· Screening was undertaken independently by two reviewers (RA and HG). Differences were discussed to determine consensus; a third reviewer (BJ) was asked to adjudicate three articles.
	Inclusion criteria	· Empirical analysis of team composition (structure) OR climate (relational processes) as the primary focus, AND staff outcomes (including job satisfaction, wellbeing, stress, or burnout) OR patient outcomes (including experience, satisfaction, or clinical effectiveness/utilisation). · Multidisciplinary team working (such as ≥2 different roles/skills). · Evidence on team composition (structure) that relates to staff ratios, grades, and profession.^11^ · Evidence on team climate that relates to relational processes of team working including discussion of shared perceptions of organisational policies, practices, and procedures.^11^
	Exclusion criteria	· Studies evaluating single roles (for example, nurses and pharmacists) or single patient groups/conditions (for example, diabetes) because they did not represent the full range of general practice service delivery.^60^ · Articles reporting change in skill mix due to task reassignment among existing team members (for example, substitution and delegation) because this was not considered to be a change in team composition.^61^ There is already a large and growing body of evidence on the effects of task reassignment.^62–65^ · Non-empirical, non-peer reviewed, grey literature, and dissertations. · Set in: secondary care, hospitals, outpatient/non-primary ambulatory care, hospices, or long-term care or home-care services.
	Quality assessment	· Two reviewers (RA and BJ) carried out independent quality assessment of all included studies using the Mixed Methods Assessment Tool (MMAT).^66^ Articles were scored (1 = high quality and well reported; 2 = good quality; and 3 = lower quality or badly reported but still relevant) so that assessments of the reviewers could be compared. In line with MMAT guidance, no studies deemed of low quality were excluded.
Data extraction	· Characteristics of included studies (bibliographic details, country of study, setting, sample/population, data and methods, variables, outcomes, and study limitations) were collated into a Microsoft Excel table (Supplementary Table S1).	
Analysis	· Texts of included articles were added to NVivo (version 12), coded, and synthesised into a thematic structure consistent with the conceptual framework and research questions.^67^ Themes were discussed with team members to corroborate findings.	

## Results

**Figure 2.**
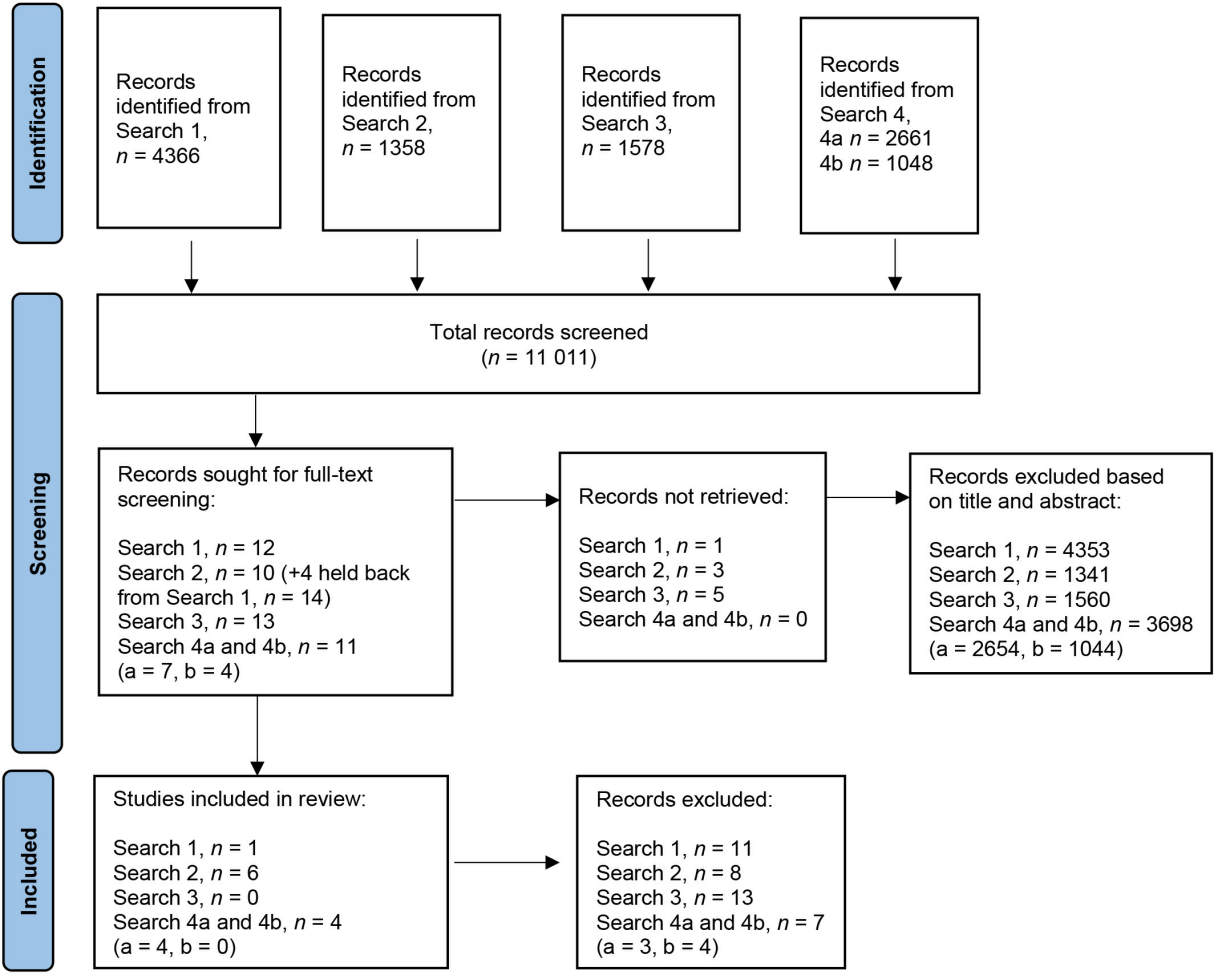
Integrated PRISMA diagram

The four searches yielded 11 011 records after de-duplication. Based on title and abstract screening, 50 records were selected for full-text screening, 39 of which were excluded because they did not explore the relationship between team composition or climate and the outcomes of interest. This resulted in 11 records for full inclusion (Figure 2 provides an integrated PRISMA covering all searches. Independent PRISMAs for each separate search can be found in Supplementary Figures S1–S4 and include exclusion reasons).

### Document characteristics

Of the 11 included studies, 10 came from the US.^18–27^ The remaining study was from Canada.^28^ All studies were empirical and used multivariate regression modelling to assess the association between composition and/or climate variables and outcomes for patients and/or staff. Data were gathered by surveys and/or from administrative data; one mixed-methods design used surveys and qualitative interviews.^25^ Of the 10 US studies, five were conducted in the Veterans Health Administration (VHA);^29–33^ three were set in other integrated healthcare systems — the Mayo clinic^18,19^ and Harvard academic collaborative;^25^ two were surveys of family physicians — one national,^20^ the other in San Francisco, US.^26^ Full data extraction tables are in Supplementary Table S2.

### Thematic summary

Studies were mapped to research questions (see Supplementary Figure S5). An overview of included articles and the measures or definitions used is in Supplementary Table S1; details of the quality assessment are in Supplementary Table S3. A summary of key findings is in Table 2.

**Table 2.** Summary of findings

	**Outcomes for staff** *The most frequently researched outcome for staff (five studies) was burnout, measured as emotional exhaustion; the predictors of work satisfaction were explored in one study*.	**Outcomes for patients** *Patient outcomes were predominantly indicators of quality of care (four studies); hospital use and all-cause mortality (indicators of the clinical effectiveness in primary health care) were used in two studies*.
**Team composition** *Mix of skills and staff characteristics (for example, team size, disciplinary mix, and provider age and* sex)	One study found emotional exhaustion or burnout was lower when physicians account for a higher proportion of the total team FTE. This study recorded higher burnout than other studies (85%) and no difference between rates for physicians and other clinicians (nurse practitioners and physician assistants).^19^ Other studies reported lower rates of burnout (30%–60%) with physicians (especially residents) at higher risk than other clinical and support staff.^21,22,26^ Two studies reported higher burnout among female physicians^19,20^ and non-physician clinicians.^19^ One study reported no association between team size and structure (family physician plus one or two or three other roles) and emotional exhaustion or burnout.^20^	Patients in practices with a predominance of female physicians reported better continuity, comprehensiveness, and responsiveness of care, and more counselling and screening, although these associations were confounded by the younger average age of female doctors. In adjusted analysis, the only significant difference from male predominant practices was worse access in female predominant practices, which was explained in terms of higher part-time working by female physicians.^28^ Hospital readmission rates were found to be lower in the panels of female clinicians, although that finding did not extend to index hospital admissions or ED visits. Panels of physicians had the lowest ED visits (versus nurse practitioners and physician assistants). The strongest predictors of higher hospital use were panel complexity and less time in clinical practice (attributed to less clinical acumen and lower risk tolerance).^18^ This study also found that hospital use was not associated with the proportion of care FTE that was physicians.
**Team climate** *The ‘relational process of teamworking’,^11^ variously measured (for example, work environment, staff stability,* *delegation, leadership, team effectiveness, team functioning, team dynamics, and workload)*	There is consistent evidence that a range of factors synonymous with good working environments and team dynamics reduce the risks of emotional exhaustion and burnout, including: team stability, staff feeling and acting like a team, having control over workload, participating in decision making, and working at the top of their competencies.^20–22,26^ Staff and skill shortages were identified as catalysts to burnout.^20–22^ One study concluded that *‘culture trumps structure*’; a poor team culture (as measured by the Team Climate Inventory) could override the effects of a stable team environment and have a negative effect on emotional exhaustion.^26^ Clinician satisfaction was associated positively with team dynamics, but through the mediation of patient care coordination: clinicians were found to derive satisfaction from better patient care coordination, which the researchers associated with good team dynamics.^25^ In another study, more than one-third of physicians reporting burnout were also satisfied.^20^	Higher workload and staff insufficiency were significantly associated with more complaints, less clinician time with patients, and lower patient-reported quality of care, with diminishing benefits observed from adding extra staff above VHA-recommended levels because of coordination problems and ‘*social loafing*’.^23,24^ Better team functioning was associated with reduced hospital admissions (vulnerable patients) and lower all-cause mortality (all patients, not vulnerable). Greater emotional exhaustion was associated with lower ambulatory care sensitive admissions; staff sufficiency was associated with higher all-cause admissions.^27^ Better relational climate and cohesion of the work group was associated with improved quality of care.^23,24^ Team climate was found to mitigate the adverse effects of high workload on patient outcomes. While workload negatively affected quality of care if the relational climate was poor, a strong relational climate can protect against poor quality of care if the workload is high.^24^ Team dynamics were found to be strongly positively associated with physician-rated patient care coordination, which, as noted above, in turn mediated a strong positive association between team dynamics and clinical work satisfaction.^25^

ED = emergency department. FTE = full-time equivalent. VHA = Veterans Health Administration

### Team composition and team climate

The impact of team composition was explored in four studies. Composition was represented by the proportion of the total primary healthcare team FTE provided by physicians,^18,19^ team size and profession,^20^ and the physician sex balance.^28^ Only one study explicitly referred to team climate.^24^ Another examined the effect of team culture, defined as ‘team functioning’ and measured using an adapted version of the Team Climate Inventory, illustrating the lack of clarity around what distinguishes the concepts of climate and culture.^26^ Team climate was indirectly implicated in several other studies through related concepts, such as team effectiveness, efficiency, and dynamics, each measured in a variety of ways; for example, communication, shared understanding, participatory decision making, and staffing stability (because of its impact on working relationships). Staff insufficiency and stressful workloads were central to several articles and linked by authors to the negative effect this has on interactions and relational work environments.^21–24,27^ Hence, staffing levels were treated as ‘climate-related’ variables in the analysis.

### Outcomes for staff

Six studies reported the association between team composition or climate and outcomes for staff, with five of these six reporting effects on emotional exhaustion or burnout^19–22,26^ and the other reporting effects on clinical job satisfaction.^25^ Associations are summarised in Table 2. Lower emotional exhaustion for all types of clinicians was associated with having a higher proportion of the total team FTE being a physician.^19^ Female clinicians were associated with a higher likelihood of burnout.^20^ Inadequate staffing^21,22^ and adverse work environments^20,21^ were associated with more emotional exhaustion. Perceived teamwork efficiency,^20^ participatory decision making,^21^ stability in team structure,^21,22,26^ and a better team ‘culture’ (measured by the Team Climate Inventory) were associated with less emotional exhaustion. Good team dynamics was strongly associated with clinician work satisfaction.^25^

### Outcomes for patients

Patient outcomes were explored in six studies, summarised in Table 2. Two focused on clinical effectiveness proxied by mortality^27^ and avoidance of unnecessary hospitalisations or emergency department visits.^18,27^ The other four focused on various care quality measures.^23–25,28^ Hospital admissions and accident and emergency visits were not associated with physician time within a care team but were predicted by greater panel complexity and fewer years in practice (less clinical acumen and lower risk tolerance). Emergency department visits were, however, lower in patient panels led by physicians than in those of physician assistants or nurse practitioners.^18^

A positive association was identified between team dynamics and patient care coordination, with the latter positively affecting clinician work satisfaction.^25^ Quality of care (influenza vaccination rates, continuity with the same practitioner, time in consultations, and patient-reported satisfaction) was worse in teams with staffing below VHA-recommended levels. However, additional staff above recommendations did not add extra benefit.^23,24^ A favourable relational climate mitigated the adverse effect of high workload on quality of care.^24^ Similarly, better team functioning, rather than staff sufficiency, was associated with lower hospital admissions.^27^

Further detail on factors affecting outcomes for staff and patients are in Supplementary Tables S4a–S4d.

## Discussion

### Summary

A central finding is that staff burnout is higher and the quality of care for patients is worse when teams are understaffed and work environments are stressful. Physicians reported higher emotional exhaustion than other clinical and non-clinical staff. One study reported less burnout when physicians accounted for a higher proportion of the whole-team FTE. Higher rates of burnout were associated with female clinicians. While having sufficient staff to afford time to patients has a beneficial effect on quality of care, additional staff may eventually have diminishing returns, which were attributed to coordination problems and ‘*social loafing*’, a term for reduced staff motivation.^23^

A stable team structure is important for effective team functioning, but less so than having a cohesive team that works well together. Indeed, a good relational climate may act as a buffer against burnout where workloads are high. Staff job satisfaction is associated with a good team dynamic and that also appears to improve patient care coordination. Varied factors were associated with lower hospital utilisation, including more years of clinical experience, less patient comorbidity, and better team functioning.

### Strengths and limitations

Despite a comprehensive search and an iterative process to widen the scope, a relatively small number of articles were identified. Searches were restricted to 10 years because the healthcare delivery landscape is constantly changing, and studies published earlier may no longer be relevant. Even so, data in two studies were from 2006.^23,24^ We also acknowledge that limiting the search to only English language articles may preclude the inclusion of valuable evidence from other countries.

Evidence from all but one study comes from the US. American provider organisations operate in a competitive environment, keep detailed patient data on service utilisation for billing, and routinely gather feedback from staff and patients to monitor their market positions, which facilitates research. Although the US studies were set in integrated healthcare systems that operate in ways similar to those of other advanced countries, including gatekeeping and rostering, context and organisation may differ such that the findings may not be directly transferable to other countries. In particular, different interpretations of what constitutes a primary healthcare team may be important. As described by articles in this review, primary healthcare practitioners in the US work with a dedicated nurse (or medical assistant) and clerk, in a small ‘teamlet’ within a primary care centre with several other ‘teamlets’. This differs from larger UK practices, where staff groupings are defined by roles. Physician-reported descriptions of teams in one study in the review revealed >800 different team compositions, indicating the challenges for researchers of analysing how staff combinations affect outcomes. The study condensed the multiple configurations to three groups (family practitioner plus one, or two, or three other roles) removing scope for nuanced interpretation.^20^

All included studies used quantitative methods (regression modelling), but cross-sectional data limited the analyses to measures of association rather than causal inference. Although the number of included studies was small, each had large sample sizes (hundreds of staff and thousands of patients). Response rates to surveys were generally >50%, except for two studies using the same dataset,^21,22^ and validated instruments (or adaptations) were used to measure climate-related variables in most studies.^21,24–27^

A narrative synthesis was necessitated by lack of consistency in the measurement of outcomes (for example, three different measures of burnout) and choice of predictor variables. There was a lack of clarity around the concept of team climate; definitions of what constitutes optimal team functioning or dynamics were varied. One study referred to team culture while measuring it using the Team Climate Inventory,^26^ others explored climate-related factors without labelling them as such. Where authors used variables reflecting relational teamworking, they were interpreted in the analysis as climate-related, but misinterpretations could have occurred. Workload was treated as climate-related because of its impact on how team members interact.^24^ Studies on climate that were relevant to the review could have been screened out because they used alternative terminology. Culture was outside of the review scope because of differences from climate in conceptualisation.^7^

### Comparison with existing literature

Since this review was conducted, two new studies have analysed national general practice workforce data in England. Use of locum (temporary) GPs was found to be higher in rural and single GP practices and was associated with inadequate performance ratings at Care Quality Commission inspections.^29^ The second study found the composition of the clinical workforce associated with various population, professional, and system outcomes in differing ways.^30,31^ While additional GPs was associated with higher satisfaction for the GPs themselves and for patients, increasing staff in other clinical roles had the opposite effect. More clinical staff was associated with better practice performance in the Quality and Outcomes Framework (QOF), but also with more hospital activity, a finding that aligns with those of a US study in the current review.^27^

In line with articles in the review, there is consensus that strong leadership, shared goals, good communication, and participatory decision making contribute to a favourable team climate and improve functioning.^12,14,32–36^ While micro-level team composition and functioning are identified as important,^37^ existing context also matters in the development of models for primary healthcare delivery and determining optimal panel sizes.^28,38,39^ Larger teams have been associated with worse scores on the Team Climate Inventory^14^ but not consistently so.^12^ Similarly, larger patient panels do not necessarily mean worse quality of care.^40^

### Implications for research and practice

Consistent with other studies,^41–44^ evidence from this review shows that in US integrated care systems in which primary healthcare practitioners (usually a physician but could be a physician’s assistant or nurse practitioner) have assigned ‘panels’ of patients, continuity of care (seeing the same practitioner) and care coordination were associated with better outcomes for staff and patients.^23,25,28^ In the NHS, concerns have been raised that combined practice lists, a so-called ‘collusion of anonymity’, result in higher utilisation and costs, increased mortality, and reduced patient satisfaction.^45^ Research is required to explore the impact of patient rostering on outcomes for patients and work satisfaction for staff, as well as the resource implications.

Studies based in the VHA report evidence-based guidelines for core primary healthcare teams regarding practitioner-to-patient ratios (900–1200 patients per physician or physicians associate or nurse practitioner, adjusted for case mix). The findings indicated patients benefit from spare capacity in a team (relative to guidelines) and diminishing returns from added staff above recommended levels.^23,24^ UK general practices make their own resourcing decisions, constrained by formula-driven practice payments that are intended to create an equitable allocation. However, the current average of 2600 patients per GP is regarded as unmanageable and has prompted the generation of guidelines for safe working.^46,47^ Investigation of optimal team sizes and economies of scale, with proposals for staff-to-patient ratios and associated incremental costs, is needed to inform decision making.^39,48,49^ With the trend for practices to increase in size, new organisational structures involving micro-teams have been suggested as a means to benefit from improved continuity of care.^44^

Studies in the review confirmed the adverse effects on staff wellbeing of insufficient staff, excessive workloads, and pressured work environments.^50,51^ These features have characterised British general practice in recent years owing to recruitment and retention problems and increased part-time working.^3,52,53^ Patient satisfaction is also at an historic low owing to access problems.^54^ Evidence from the review supports the mitigating impact of a good team climate on the adverse effects of high workload.^24^ However, more clarity is required on what ‘good’ looks like, the factors that create it, and how these are generated. Articles in the review variously indicate the importance of goals, leadership, and inclusivity for promoting productive interactions. Research is now needed to identify a clear conceptualisation of team climate specific to a healthcare context, which will inform the development of interventions to improve working environments.

There was little evidence from the review to inform the current policy of introducing additional roles into general practice to address staff shortages. Further research is required to explore whether having adequate staff per se or a greater variety of roles is the more effective at reducing work pressure and improving patient experiences,^31^ and how part-time staff affiliations in practices affects team climate. Similarly, studies in the review do not directly inform the post-pandemic debate about how use of remote consultation methods affects the quality of care,^45^ although they provide consistent evidence of the importance to patients of good access and a personalised approach. They also indicate that allowing patients more time with practitioners improves quality of care and patient satisfaction,^24^ which supports recent recommendations for increasing the length of consultations in general practice,^55^ with consultation lengths in the UK currently being the briefest in Europe.^56^

Finally, the increasing numbers of females becoming GPs also requires consideration. The present review, and another,^57^ have suggested that females may be at greater risk of emotional exhaustion,^19,20^ and that their higher rates of part-time working may adversely affect access and continuity of care for patients.^28^ While the new wellbeing QOF indicator is intended to reduce GP burnout, it places a significant onus on individual practices and individuals themselves.^58^ Policies and guidance are urgently needed to support local initiatives.^59^ In particular, how the working environment affects females and their ability to achieve wellbeing, job satisfaction, and deliver patient care now requires further research.
